# Alginate-based 3D cell culture technique to evaluate the half-maximal inhibitory concentration: an in vitro model of anticancer drug study for anaplastic thyroid carcinoma

**DOI:** 10.1186/s13044-021-00118-w

**Published:** 2021-12-03

**Authors:** Hilda Samimi, Alireza Naderi Sohi, Shiva Irani, Ehsan Arefian, Mojdeh Mahdiannasser, Parviz Fallah, Vahid Haghpanah

**Affiliations:** 1 Endocrinology and Metabolism Research Center, Endocrinology and Metabolism Clinical Sciences Institute, Tehran University of Medical Sciences, Tehran, Iran; 2 Department of Biology, Science and Research Branch, Islamic Azad University, Tehran, Iran; 3 Department of Nanotechnology and Tissue Engineering, Stem Cell Technology Research Center, Tehran, Iran; 4 Molecular Virology Lab, Department of Microbiology, School of Biology, College of Science, University of Tehran, Tehran, Iran; 5 Department of Laboratory Science, Faculty of Allied Medicine, Alborz University of Medical Sciences (ABZUMS), Taleghani Boulevard, Taleghani Square, Karaj, 3155717453 Iran; 6 Personalized Medicine Research Center, Endocrinology and Metabolism Clinical Sciences Institute, Tehran University of Medical Sciences, Tehran, Iran

**Keywords:** Anaplastic thyroid carcinoma, Two-dimensional cell culture, Three-dimensional cell culture, Anticancer drug, Half-maximal inhibitory concentration

## Abstract

**Background:**

Three-dimensional (3D) cell culture methods are identified for simulating the biological microenvironment and demonstrating more similarity to in vivo circumstances. Anaplastic thyroid carcinoma (ATC) is a lethal endocrine malignancy. Despite different treatment approaches, no improvement in the survival rate of the patients has been shown. In this study, we used the 3D in vitro ATC model to investigate the cytotoxic effect of BI-847325 anticancer drug in two-dimensional (2D)- and 3D- cultured cells.

**Methods:**

Human ATC cell lines, C643 and SW1736, were cultured in one percentage (w/v) sodium alginate. Spheroids were incubated in medium for one week. The reproducibility of the fabrication of alginate beads was evaluated. Encapsulation of the cells in alginate was examined by DAPI (4^′^,6-diamidino-2-phenylindole) staining. Survival of alginate-encapsulated cells was evaluated by CFSE (5,6-Carboxyfluorescein N-hydroxysuccinimidyl ester) staining. The population doubling times of C643 and SW1736 cell lines cultured in 2D monolayer as well as in 3D system were calculated. The cytotoxic effect of BI-847325 on 2D- and 3D- cultured cell lines was assessed for 24–72 h by MTT [3-(4,5-dimethylthiazolyl-2)-2,5-diphenyltetrazolium bromide] assay. Finally, the 3D culture results were compared with the 2D culture method.

**Results:**

The half-maximal inhibitory concentration (IC_50_) values of BI-847325 were higher in 3D culture compared to 2D culture. The cytotoxicity data indicated that 3D in vitro models were more resistant to chemotherapy agents.

**Conclusions:**

The findings of this study are beneficial for developing in vitro ATC 3D models to analyze the efficacy of different chemotherapy drugs and formulations.

## Introduction

Thyroid cancer is the most frequent endocrine malignancy with the highest growing incidence rate between the other types of solid tumors in the United States [1–3]. Anaplastic thyroid carcinoma (ATC), also known as undifferentiated thyroid cancer (UTC), has one of the worst prognoses among other types of thyroid cancer, mainly due to its aggressive behavior and resistance to chemotherapy agents [4]. Despite different cancer treatment approaches, there is no improvement in the survival rate of patients [5]. In spite of the basic differences between 2D conditions and three dimensional (3D) cell environments, merely found in vivo, the majority of in vitro ATC cell culture experiments are performed via monolayer (2D) cells cultured on flask or dish surfaces. Conventional 2D-grown cells have served researchers well for decades, but this culture model is a non-natural system that does not represent the natural microenvironment [6, 7].

In general, cancer investigators rely on 2D cell culture in vitro experiments and animal models to understand the complex biological processes of tumor formation, progression and, its treatment [8, 9]. In recent years, more biologists have started using 3D culture systems, which provide environments more similar to their physiological conditions. 3D cell culture models more closely mimic key factors of natural tumor microenvironment such as molecular concentration gradients and crucial cellular processes such as cell to cell and cell to extracellular matrix (ECM) interactions. These culture systems act as a surrogate for animal models and are less expensive and provide quicker results compared for animal models [10–12]. Therefore, 3D cell culture systems can play an important role in anticancer drug discovery due to the lack of animal models including lower primates or fewer animal models in rare cancers such as ATC, ethical considerations, and advantages greater than 2D cell culture [13].

Scaffold-based 3D cell culture systems are increasingly becoming necessary tools in cancer research [14]. Some hydrogels are polymers that because of their hydrophilic nature, keep high amounts of water following gelation. Hydrogels have high water retention ability due to interconnected microscopic pores, which facilitate easy transport of O_2_, nutrients, metabolic wastes, growth factors, etc. Therefore, hydrogel scaffolds are used in different pharmaceutical and biomedical fields [6]. Alginate, a natural polymer with the ability to create hydrogel, is known for properties such as its ability to allow diffusion of nutrients and other medium compositions including chemotherapy drugs [6].

Drug discovery and development is still a slow and costly process with little success in clinical trials. At present, more than 50% of all medicines fail due to lack of effectiveness in Phase II and III clinical trials [12]. Also, just 12% of drugs that enter clinical trials are ultimately approved to be applied in humans. The importance of in vitro cell culture models is the requirement to more cost-effective advancement of novel chemotherapy drug discovery and time-effective treatment of cancers [15]. The promising methods that are expected to improve drug discovery and development success rate are new preclinical models that better mimic in vivo microenvironmental factors. It is now widely accepted that culturing cells in 3D systems better represents the natural microenvironmental conditions than simple 2D monolayer methods due to more accurate simulation of key tissue factors [16–20]. More efficient drugs are probably developed using more reliable cell culture systems. Therefore, suitable cell culture systems would additionally decrease the requirement for animal trials, particularly for drug toxicity assays [21]. However, the importance of animal models cannot be underestimated in biochemical and physiological studies, cancer research, and drug screening [22].

Recently, scientists have begun studying cancer stem cells (CSCs) in 3D cell culture models and altering culture elements in order to better imitation of in vivo conditions [23, 24]. Culturing CSCs in 2D may reduce their stemness properties, while CSCs cultured in 3D show more resistance against anticancer drugs [23]. Now, there is no doubt that 3D cell culture systems are biologically further applicable in the field of cancer research because they can provide cellular responses that need more biological communication. At the present, thyroid CSCs have been investigated, especially as the origin of ATC. This subpopulation of cells is responsible for resistance to chemotherapy drugs. Targeted therapy against CSCs has improved ideas in the field of ATC treatment and regenerative chemotherapy drugs [25]. Indeed, MAPK signaling and aurora kinase family are now becoming clear to be involved in thyroid tumorigenesis, particularly in ATC [26]. In the clinic, selective MEK and aurora kinase inhibitors are associated with high levels of response in ATC patients [27]. BI-847325, as a multi-target anticancer drug, selectively and simultaneously inhibits MEK and aurora kinases [28]. Therefore, it can be considered as a candidate for chemotherapy drugs in the treatment of ATC.

In this study, we used the alginate-based 3D in vitro ATC model as an initiative to investigate the differences between half-maximal inhibitory concentration (IC_50_) of dual MEK/Aurora kinase inhibitor BI-847325 on 2D- and 3D- cultured ATC C643 and SW1736 cell lines.

## Material and methods

### 2D cell culture

Human ATC cell lines, C643 and SW1736, were acquired from CLS Cell Lines Service GmbH, Germany. Both of the cell lines were cultured in RPMI 1640 medium (Invitrogen, Carlsbad, CA, USA) supplemented with 10% fetal bovine serum (Invitrogen, Carlsbad, CA, USA), 2 mM glutamine (Sigma-Aldrich, St. Louis, MO, USA), 100 units of penicillin/ml (Sigma-Aldrich, St. Louis, MO, USA), and 100 mg of streptomycin/ml (Sigma-Aldrich, St. Louis, MO, USA). The cells were incubated in 5% CO_2_ at 37 °C.

### 3D cell culture

One percentage (w/v) sodium alginate (Sigma-Aldrich, St. Louis, MO, USA) was prepared in HEPES buffer (Merck Millipore, UK, 20 mM + NaCl 155 mM, pH 7.4). CaCl_2_ solution (Merck Millipore, UK, 102 mM) was prepared in HEPES buffer. C643 and SW1736 cells were resuspended in sodium alginate at 2 × 10^5^ viable cells/ml. The alginate-cell suspension was extruded through a 22-G needle into the CaCl_2_ solution. The resultant alginate spheroids remained in the CaCl_2_ solution for 10 min at room temperature to induce cross-linking within the alginate. The spheroids were subsequently washed with PBS and then RPMI 1640 with 10% FBS. Afterward, cells were cultured in alginate spheroids for one week.

### Encapsulation and survival of cells in sodium alginate

3D spheroids of ATC cell lines were prepared using the sodium alginate hydrogel as described above. The obtained spheroids were imaged by a camera (Leica) followed by measuring their size using ImageJ software (NIH). The probability plot for the measured sizes was constructed by Minitab v16 software with a confidence interval of 95%. In the next step, spheroids were washed with PBS and stained separately with DAPI (4^′^,6-diamidino-2-phenylindole) (Sigma-Aldrich, St. Louis, MO, USA) and CFSE (5,6-Carboxyfluorescein N-hydroxysuccinimidyl ester) (Invitrogen, Carlsbad, CA, USA) as described by the manufacturer and imaged with a fluorescence microscope (Nikon TE2000-S).

### Calculation of population doubling time

In the next step, population doubling times of C643 and SW1736 cell lines cultured in 2D monolayer as well as in 3D system were obtained by using the following formula (http://www.doubling-time.com/compute.php) and compared:

$$\mathrm{Doubling}\ \mathrm{Time}=\frac{\mathrm{Duration}\times \log (2)}{\log \left(\mathrm{final}\ \mathrm{concentration}\right)-\log \left(\mathrm{initial}\ \mathrm{concentration}\right)}$$

### Cell viability assay

#### 2D cell culture

The effect of BI-847325 (Adooq Bioscience) on cell viability was assessed by MTT [3-(4,5-dimethylthiazolyl-2)-2,5-diphenyltetrazolium bromide] assay. The C643 and SW1736 cell lines were seeded in 96-well plates at a concentration of 5000 cells/well. After 24 h cells were treated with 0.125–8 μM doses of BI-847325 for 24–72 h. The viability of the cells was evaluated by the metabolic conversion of the tetrazolium dye to insoluble formazan. MTT (5 mg/ml) was added to the 96-well plates at 10 μl/well, and the plates were then incubated for three hours in 5% CO_2_ at 37 °C incubator. Finally, the formazan crystals were solubilized in 100 μl/well DMSO.

#### 3D cell culture

The effect of BI-847325 treatment on encapsulated ATC cell lines was also examined by MTT assay. Cells were treated with BI-847325 (1–64 μM) for 24 h as described earlier [29], and currently for 48 h and 72 h with the same doses. MTT solution (5 mg/ml) was added to each well of a 48-well plate at 10 μl/well. Encapsulated cells were incubated for 4 h in 5% CO_2_ at 37 °C incubator. The medium was removed cautiously with a pipette to avoid the disruption of beads. To dissolve the formazan salt, 500 μl of 0.1 M HCl (Merck Millipore, UK) in 2-propanol (Merck Millipore, UK) was added. Plates were placed in the incubator for 45 min to allow the formazan to diffuse out of the alginate hydrogel and entirely be dissolved in the solvent [30]. The absorbance of the solution was measured by ELISA reader (BioTek ELx800) at 570 nm. Each experiment was performed in quadruplicate to assess the consistency of results. IC_50_ values were calculated via the Reed–Muench method [31, 32].

### Statistical analysis

The data show the mean of four independent experiments±SD. Statistical analysis of MTT results were performed using One-way ANOVA followed by Tukey’s post hoc tests. Statistical significances are expressed as *p* < .05 (*); *p* < .01 (**); *p* < .001 (***); *p* < .0001 (****). IC_50_ values were calculated by nonlinear regression using GraphPad Prism v5 software. Statistical comparisons of population doubling times were carried out using the unpaired t-test method in each case (*n* = 3).

## Results

### Encapsulation and survival of cells in sodium alginate

**Fig. 1:**
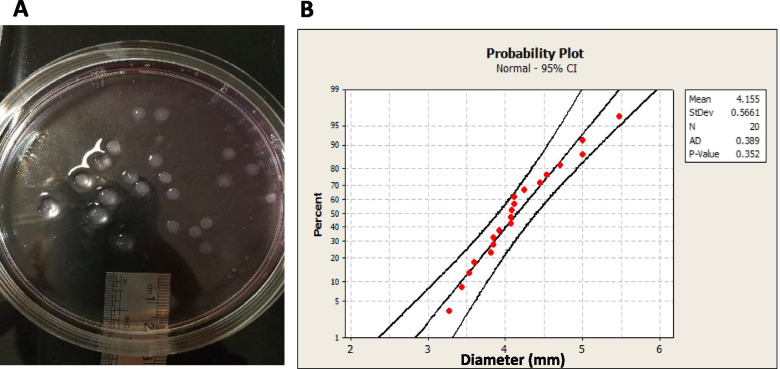
**Determination the size of spheroids.** (**A**) A petri dish containing alginate spheroids was imaged to measure the size of spheroids using ImageJ software (NIH) (*n* = 20). (**B**) Probability plot for the measured sizes. Confidence interval was adjusted to 95%. *P*-value was obtained as .352

**Fig. 2:**
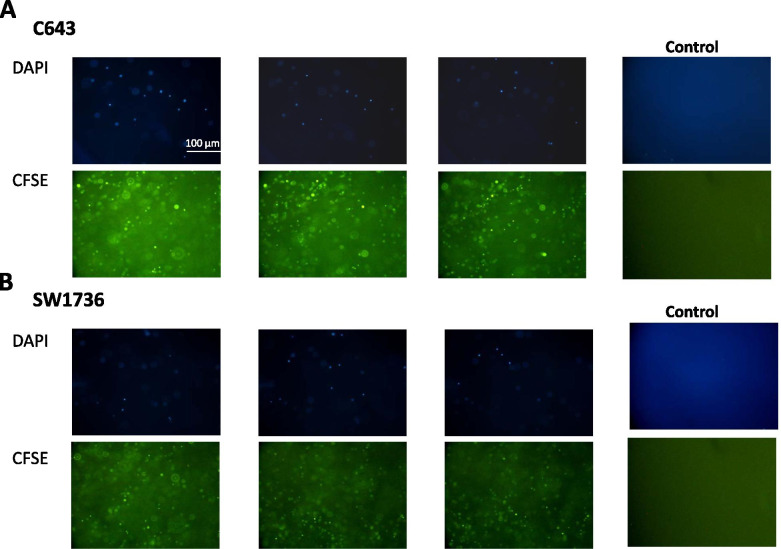
**Encapsulation and survival of ATC cell lines in sodium alginate.** Alginate-encapsulated (**A**) C643 and (**B**) SW1736 ATC cell lines were separately stained with DAPI and CFSE for and imaged with fluorescence microscope. In each case, three images taken with different focus distances are shown. A cell-free alginate scaffold was used as a control in each group

First, to evaluate the reproducibility of the fabrication of alginate spheroids (*n* = 20), their size was measured using ImageJ software (Fig. 1A). According to the probability plot (Fig. 1B), the size of spheroids had a normal distribution (*P*-value = .352) which indicates appropriate reproducibility in spheroids formation. Encapsulation and survival of cell lines are shown in Fig. 2.

### Determination of population doubling time

**Fig. 3:**
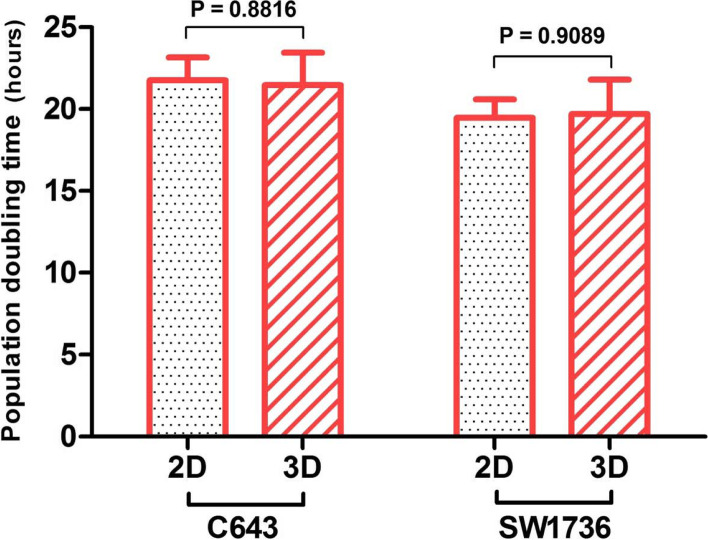
**Population doubling time of C643 and SW1736 cell lines in 2D and 3D culture.** n = three independent experiments. *P*-values were obtained by unpaired t-test method in each case and are represented in graph

As shown in Fig. 3, the difference between the population doubling times of cells cultured in 2D and 3D was not statistically significant (*p* value>.05) indicating the same growth rate of ATC cell lines in 2D and 3D cultures.

### Determination of IC_50_ in the 2D cell culture system

**Fig. 4:**
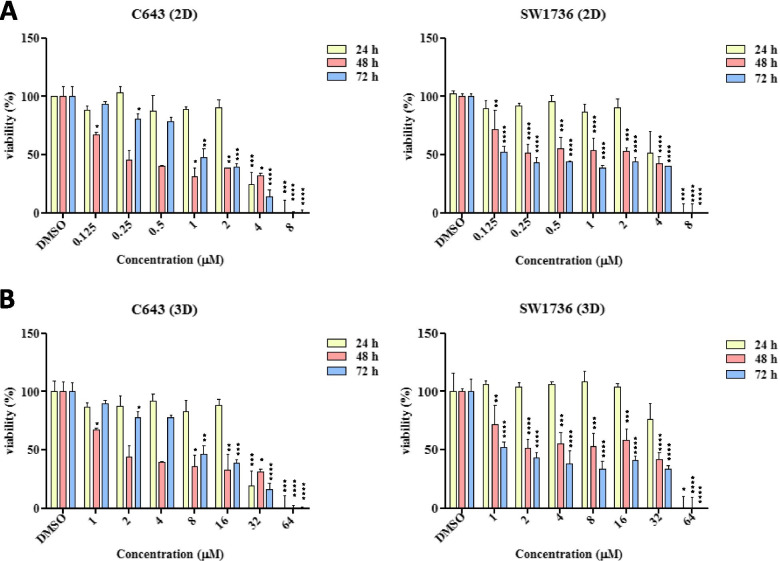
**Inhibitory effects of BI-847325 on viability of ATC cell lines.** C643 and SW1736 cell lines were treated with the indicated concentrations of BI-847325 in (**A**) 2D and (**B**) 3D cell culture systems for 24–72 h, followed by MTT assay to evaluate cell viability. IC_50_ values of BI-847325 for each cell line in two cell culture systems were calculated according to the Reed-Muench methods. The viability of the cells treated by different concentration of drug was normalized to that of untreated ones which were exposed to DMSO with the concentration equal to what existed in drug solutions. This normalization process was performed for each time of assay (i.e. 24, 48, and 72 h) separately. For each time point, the viabilities of the cells treated with different concentration of BI-847325 were compared and statistically analyzed via One-way ANOVA and Turkey’s post-test method. The significant differences comparing the control (i.e., untreated) cells are represented by star(s) as follows: *p < .05, **p < .01, ***p < .001, ****p < .0001)

Evidence has shown that through carefully selecting thyroid cancer cell lines, an appropriate in vitro model can be developed to analyze the signaling pathways associated with thyroid carcinogenesis [33]. In this study, ATC cell lines with different genetic backgrounds in the MAPK signaling pathway were used, C643 with *RAS* mutation and SW1736 with *BRAF*^V600E^ mutation [33–36]. To evaluate the effect of BI-847325 on the viability of these cell lines, we evaluated the concentration-response and time course of BI-847325 in two selected cell lines. As shown in Fig. 4A, inhibition of cell proliferation occurred in both cell lines in a concentration-dependent manner. The treatment panel of the cell lines with BI-847325 (0.125–8 μM) for 24-72 h prevented proliferation in two cell lines. In both cell lines, IC_50_ remained constant after 48 h and IC_50_ values of BI-847325 on C643 and SW1736 were at 2 μM and 4 μM, respectively (Table 1). The IC_50_ values demonstrated that C643 cell line was twice as sensitive to BI-847325 compared to the SW1736 cell line.

**Table 1:** Differences between IC_50_ values of BI-847325 in 2D and 3D ATC cell culture systems

Cell lines	Hours	IC_**50**_	
		**2D**	**3D**
**C643**	24	3 μM	25 μM
	48	2 μM	15 μM
	72	2 μM	15 μM
**SW1736**	24	5 μM	43 μM
	48	4 μM	34 μM
	72	4 μM	34 μM

### Determination of IC_50_ in the 3D cell culture system

In this study, we examined the effect of dual MEK/Aurora kinase inhibitor BI-847325 on the viability and proliferation of C643 and SW1736 cell lines that were cultured in 3D alginate spheroids. We found that BI-847325 significantly inhibited the proliferation of these cell lines. This inhibition occurred in a concentration-dependent manner. The treatment panel of the C643 and SW1736 cell lines with BI-847325 (1–64 μM) for 24–72 h prevented proliferation in the two cell lines (Fig. 4B). In both cell lines the growth-inhibitory effect of BI-847325 remained constant after 48 h and IC_50_ values of BI-847325 on C643 and SW1736 were at 15 μM and 34 μM, respectively (Table 1).

Interestingly, similar to 2D cell culture, the IC_50_ values in the 3D method demonstrated that C643 cell line is more sensitive towards BI-847325 compared to the SW1736 cell line.

## Discussion

**Fig. 5:**
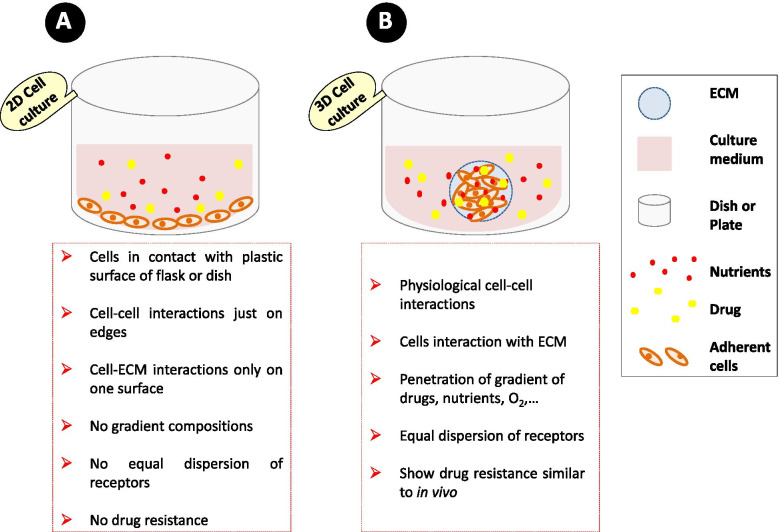
**Comparison of 2D and 3D cell culture systems. (A)** 2D cell culture, (**B**) 3D cell culture

The results of this study are the first report of 3D model for a more accurate assessment of the effect of chemotherapy drugs in ATC. These findings indicate that 3D in vitro models demonstrate more resistance to anticancer drugs. Increased IC_50_ values in 3D cell culture system may be associated with multiple mechanisms, including a reduced penetration of chemotherapy agents because of the simulation of key factors of natural tumor microenvironment such as physiological gradients and presence of ECM, enhanced pro-survival signaling pathways such as MAPK pathway due to upregulation of genes involving in anticancer drug resistance [19, 37–41] (Fig. 5).

Furthermore, CSCs cultured in the 2D system may reduce their stemness characteristics, while CSCs cultured in 3D may be more resistant to anticancer drugs [19, 23, 42, 43]. Considering the benefits of 3D cell culture systems, mainly in cancer research, we used an in vitro ATC cell culture model by applying a biologically inert alginate hydrogel scaffold for determining the effective dose of dual MEK/Aurora kinase inhibitor BI-847325.

BI-847325 cytotoxicity on ATC C643 and SW1736 cell lines was measured in 2D and 3D cell culture systems. The study has revealed that the concentration ranges used in the 2D cell culture method were not appropriate to exhibit significant cytotoxicity in the 3D cell culture system [9]. Based on this evidence, the concentration range of 1–64 μM was selected for the determination of the effective dose of BI-847325 anticancer drug in the 3D cell culture method. As anticipated and evidenced in the initial investigation, the IC_50_ values were significantly higher in the 3D cell culture system compared to the 2D one for both cell lines (Table 1). The cytotoxicity assay showed that IC_50_ values of BI-847325 for C643 and SW1736 were at 15 μM and 34 μM concentrations, respectively. The higher IC_50_ values in 3D cell cultures demonstrate the resistance potential gained by cells while growing in the 3D system which simulates the natural physiological conditions. This evidence supports the higher resistance rate of cultured cells in the 3D system to chemotherapy drugs as reported previously [9, 23].

In a similar study, higher viability rates following treatment with Paclitaxel were observed in ovarian cancer cells cultured in 3D compared to 2D cell culture [44]. In another study, comparison of 3D and 2D tumor models in breast cancer was also revealed higher human epidermal growth factor receptor 2 (HER2) activation in the 3D model associated with the less response to Trastuzumab [45]. Therefore, the determination of chemosensitivity in 2D cell culture systems cannot reflect tumor drug sensitivity. These findings indicate that chemotherapy drugs evaluation in the 3D cell culture system may provide useful achievement that is not accessible in conventional 2D investigations.

On the other hand, the subpopulation of malignant cells in the tumor, known as CSCs, is responsible for cancer recurrence and different drug resistance mechanisms [46–48]. We have evaluated the BI-847325 cytotoxicity on ATC C643 and SW1736 cell lines with the CSCs characteristics [49] in two different cell culture models and found that the IC_50_ values are higher in 3D cell culture system compared to the 2D cell culture method. Like what observed in 2D culture, the obtained IC_50_ in the 3D culture for SW1736 was higher than that for C643 similar to conventional 2D culture. This observation indicates the resistant nature of this cell line which is probably due to more stemness properties of ATC SW1736 cell line. The elevated IC_50_ values confirm the Lei and colleagues report about 3D matrix-driven CSCs theory in which 3D culture of breast cancer cell line, MCF-7, resulted in more tumorigenesis and elevated anticancer drug resistance [9, 23, 50].

In order to understand the difference between cytotoxic responses of chemotherapeutic drugs in 2D- and 3D- based culture systems, Godudu et al. examined the uptake efficiency of Doxorubicin inside the alginate-encapsulated lung cancer cell lines [9]. Doxorubicin uptake examination showed that only a low amount of accessible drug (~ 10%) was penetrated into the spheroids. This investigation confirmed the poor permeability of tumors [9]. Because of poor penetration, a high concentration of chemotherapy agents is needed to demonstrate cytotoxicity in tumor cells [43, 51, 52]. This poor penetration of drugs is revealed by several similar models applying 3D cell culture systems based on spheroids [37, 53].

Collectively, 3D cell culture systems such as alginate-encapsulated cells more closely mimic the key factors of the natural tumor microenvironment such as molecular concentration gradients and important cellular interactions including cell-cell and cell-matrix communications. However, additional cellular investigations such as xenograft tumors are required to promote the predictive value of 3D cell culture in the response of ATC cells to anticancer drugs in vivo.

## Conclusion

In this study, the cytotoxicity data indicated that 3D in vitro models display more resistance to chemotherapy agents. ATC tumors are unresponsive to the current anticancer drugs. Having to do with 3D in vitro ATC models designed by the current research, it would be feasible to analyze the effect of different anticancer drugs and diverse signaling pathways affected by the chemotherapy agents and formulations. In terms of the future application of the proposed model, this culture system can be modified to support the multicellular 3D cell culture. Moreover, the attachment of different peptides can improve alginate scaffold according to extracellular matrix properties.

## Data Availability

All data generated or analyzed during this study are included in this published article.

## Abbreviations

2D: two dimensional
3D: three dimensional
ATC: anaplastic thyroid carcinoma
CFSE: 5,6-Carboxyfluorescein N-hydroxysuccinimidyl ester,
CSC: cancer stem cell
DAPI: 4^′^,6-diamidino-2-phenylindole
ECM: extracellular matrix
ELISA: enzyme-linked immunosorbent assay
HEPES: N-2-hydroxyethylpiperazine-N-ethanesulfonic acid
HER2: human epidermal growth factor receptor 2
IC_50_: half-maximal inhibitory concentration
MEK: Mitogen-activated protein kinase kinase
MTT: 3-(4,5-dimethylthiazolyl-2)-2,5-diphenyltetrazolium bromide
UTC: undifferentiated thyroid cancer.
